# Temporal Trends in Fast-Food Restaurant Energy, Sodium, Saturated Fat, and *Trans* Fat Content, United States, 1996–2013

**DOI:** 10.5888/pcd11.140202

**Published:** 2014-12-31

**Authors:** Lorien E. Urban, Susan B. Roberts, Jamie L. Fierstein, Christine E. Gary, Alice H. Lichtenstein

**Affiliations:** Author Affiliations: Lorien E. Urban, Susan B. Roberts, Jean Mayer USDA Human Nutrition Research Center on Aging, Tufts University, Boston, Massachusetts; Jamie L. Fierstein, Christine E. Gary, Freidman School of Nutrition Science and Policy, Tufts University, Boston, Massachusetts.

## Abstract

**Introduction:**

Excess intakes of energy, sodium, saturated fat, and *trans* fat are associated with increased risk for cardiometabolic syndrome. Trends in fast-food restaurant portion sizes can inform policy decisions. We examined the variability of popular food items in 3 fast-food restaurants in the United States by portion size during the past 18 years.

**Methods:**

Items from 3 national fast-food chains were selected: French fries, cheeseburgers, grilled chicken sandwich, and regular cola. Data on energy, sodium, saturated fat, and *trans* fat content were collated from 1996 through 2013 using an archival website. Time trends were assessed using simple linear regression models, using energy or a nutrient component as the dependent variable and the year as the independent variable.

**Results:**

For most items, energy content per serving differed among chain restaurants for all menu items (*P* ≤ .04); energy content of 56% of items decreased (β range, −0.1 to −5.8 kcal) and the content of 44% increased (β range, 0.6–10.6 kcal). For sodium, the content of 18% of the items significantly decreased (β range, −4.1 to −24.0 mg) and the content for 33% increased (β range, 1.9–29.6 mg). Absolute differences were modest. The saturated and *trans* fat content, post-2009, was modest for French fries. In 2013, the energy content of a large-sized bundled meal (cheeseburger, French fries, and regular cola) represented 65% to 80% of a 2,000-calorie-per-day diet, and sodium content represented 63% to 91% of the 2,300-mg-per-day recommendation and 97% to 139% of the 1,500-mg-per-day recommendation.

**Conclusion:**

Findings suggest that efforts to promote reductions in energy, sodium, saturated fat, and *trans* fat intakes need to be shifted from emphasizing portion-size labels to additional factors such as total calories, frequency of eating, number of items ordered, menu choices, and energy-containing beverages.

## Introduction

Excess intakes of energy, sodium, saturated fat, and *trans* fat are associated with elevated risk for cardiometabolic disorders (1–3). For this reason, Dietary Guidelines for Americans (1) and health advocacy organizations (4–6) recommend limiting intakes and maintaining a healthy weight. Nevertheless, intakes of these nutrients exceed recommendations (1–7).

The contribution of away-from-home foods to total energy has nearly doubled in the past 30 years, rising from 18% in 1977 to 33% in 2010 (8,9), and fast food in particular has historically contributed a disproportional amount of dietary sodium, saturated fat, and *trans* fat (10–12), making these foods a target for modification. Although there has been progress in this area, including an increase in the number of “healthier” offerings, sales for the most frequently ordered items from fast-food restaurants remain strong (15).

One area that has gained attention is the portion size (ie, amount served to customer) of frequently ordered items. Between 1998 and 2006, fast-food retailers attempted to minimize publicizing the issue of changing portion sizes by redesignating sizing (eg, medium renamed small), which resulted in an increase of portion sizes in absolute terms (13). Little information exists for trends in the energy content of fast-food items since 2006 or trends in the amounts of sodium, saturated fat, and *trans* fat in fast-food menu items over time. These data are important because, in addition to changes in menu options, they can be used as an indicator of whether foods as served outside the home have been modified to be consistent with population-wide dietary guidance. They also provide a basis on which to evaluate industry trends and provide data to inform public health campaigns and clinical programs designed to promote improvements in dietary patterns.

Our aim was to collate available data for energy, saturated fat, *trans* fat, and sodium for some of the most frequently ordered fast-food items from 3 national fast-food chains by portion size and describe trends over a 18-year period from 1996 through 2013.

## Methods

Three fast-food chain restaurants (designated Chain A, Chain B, and Chain C) were selected as examples on the basis of their offering similar menu items, having a national presence, and being in the top 10 for total US sales revenue (14). Chain A was identified as the top restaurant on the basis of sales; the other restaurants were then chosen according to the criteria described above. The most commonly ordered menu items offered according to a recent report (15) included French fries (fried potatoes; small, medium, and large), cheeseburger (approximately 2 oz and 4 oz, uncooked beef weight), grilled chicken sandwich (1 available size), and regular cola drink (small, medium, and large). To obtain objective and complete information, the Wayback Machine (http://www.archive.org/web/web.php) was used to collate data for energy (kcal/portion), sodium (mg/portion), saturated fat (g/portion), and *trans* fat (g/portion). The Wayback Machine is a publicly available web archive database that includes information, in this case, from company websites. For the 4% of data not available from the Wayback Machine website, nutrition information was obtained directly from restaurant websites or found at other Internet sites. 

Our analysis included 3 food items for energy, sodium, saturated fat, and *trans* fat; 1 beverage item was included in the energy analysis only, because the sodium content of cola beverages is low and may vary by local water supply, and the beverage did not contain fat. Because fast-food restaurant orders and special offers frequently include a cheeseburger, French fries, and regular cola (bundled meal), we assessed the combined energy, sodium, saturated fat, and *trans* fat amounts and calculated the relative contribution of each in each chain for small and large portions.

Time trends were assessed for energy, sodium, saturated fat, and *trans* fat per serving for individual menu items at each chain using simple linear regression models in which energy content or the nutrient was the dependent variable and year was the independent variable. Differences among chains for individual menu items were assessed using analysis of variance for the mean energy or nutrient components across the 18-year period, and the Tukey post hoc procedure was used to control for multiple comparisons. Statistical analyses were performed using SAS 9.3 (SAS Institute Inc).

## Results

### Individual menu items

#### Energy

**Figure 1:**
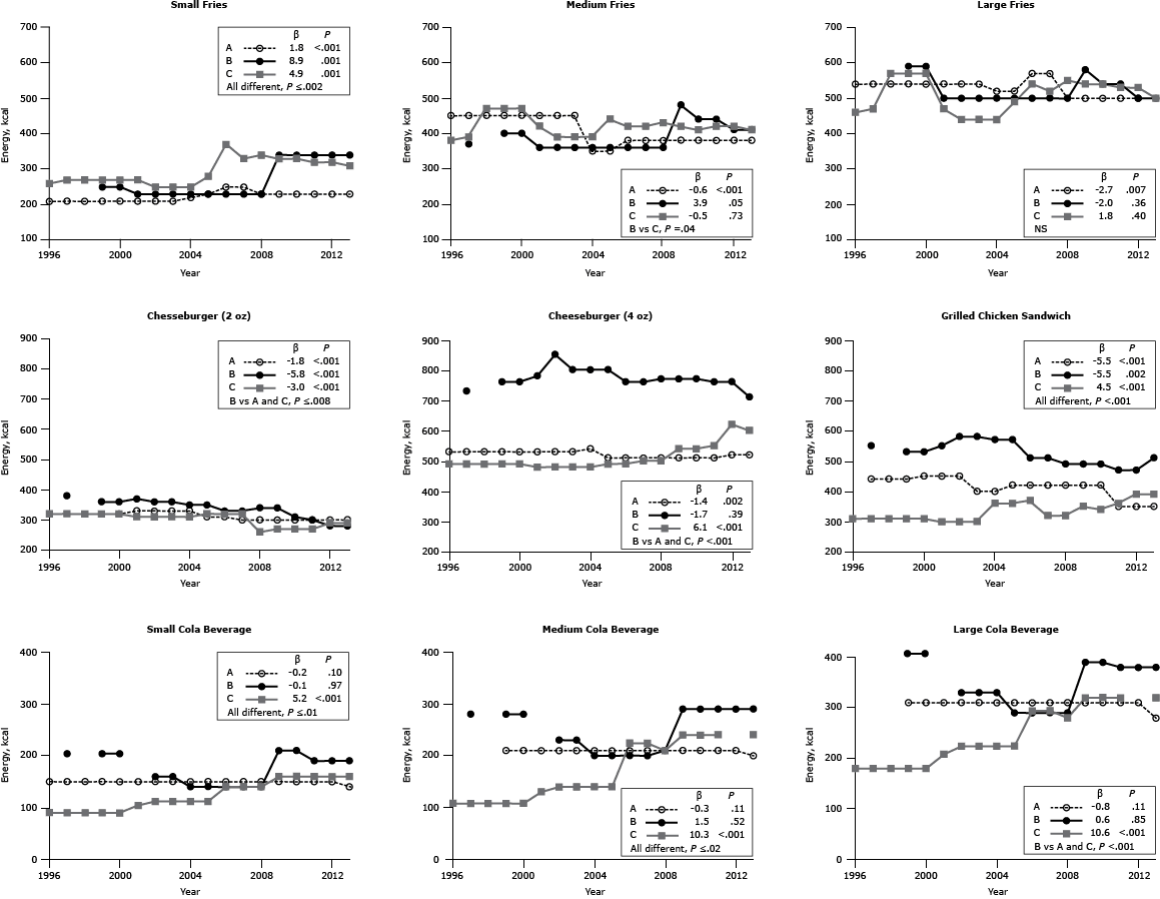
Energy content (kcal per portion) for popular menu items at 3 large, national fast-food chains. Energy content for 3 sizes of French fries (small, medium, large); 2 sizes of cheeseburgers (2 oz, 4 oz); 1 size of grilled chicken sandwich; and 3 sizes of cola beverages (small, medium, large) from chains A, B, and C from 1996 through 2013. β estimates and *P *values derived from individual simple linear models; chain comparison *P* values derived from ANOVA (analysis of variance) models comparing mean values between restaurants. Dashes indicate that data were not available; blank cells indicate that the item was not offered for the year(s). Abbreviations: S, small; M, medium; L, large; NS, nonsignificant. ^a^ Difference is between Chain B versus Chain C. ^b^ Difference is between Chain B versus Chains A and C. **Table:** 

The energy content of the 27 items examined differed among chain restaurants for all menu items (*P* ≤ .04) except for large French fries. The energy content of 15 items (56%) decreased over the 18-year period (β range, −0.1 to −5.8 kcal); of these, the differences were significant for 8 items (30%; β range, −0.6 to −5.8 kcal, *P* < .01) (Figure 1). For items whose energy content decreased, 5 items were offered at Chain A, 2 items at Chain B, and 1 item at Chain C. The energy content of 12 items (44%) increased over the 18-year period (β range, 0.6–10.6 kcal); of these, the differences were significant for 9 items (33%) (β range, 1.8–10.6 kcal, *P* ≤ .05). For items whose energy content increased, 6 items were offered at Chain C, whereas Chain A offered 1 item and Chain B offered 2 items. In absolute terms, a similar number of items increased and decreased in energy content, and the mean changes were modest.

Chain/Year	French Fries, kcal			Cheeseburger, kcal		Grilled Chicken Sandwich, kcal	Cola, kcal		
	S	M	L	2 oz	4 oz		S	M	L
**Chain A**									
1996	210	450	540	320	530		150	—	—
1997	210	450	540	320	530	440	150	—	—
1998	210	450	540	320	530	440	150	—	—
1999	210	450	540	320	530	440	150	210	310
2000	210	450	540	320	530	450	150	210	310
2001	210	450	540	330	530	450	150	210	310
2002	210	450	540	330	530	450	150	210	310
2003	210	450	540	330	530	400	150	210	310
2004	220	350	520	330	540	400	150	210	310
2005	230	350	520	310	510	420	150	210	310
2006	250	380	570	310	510	420	150	210	310
2007	250	380	570	300	510	420	150	210	310
2008	230	380	500	300	510	420	150	210	310
2009	230	380	500	300	510	420	150	210	310
2010	230	380	500	300	510	420	150	210	310
2011	230	380	500	300	510	350	150	210	310
2012	230	380	500	300	520	350	150	210	310
2013	230	380	500	300	520	350	140	200	280
**β**	1.8	−0.6	−2.7	−1.8	−1.4	−5.5	−0.2	−0.3	−0.8
***P* value**	<.001	<.001	.007	<.001	.002	<.001	.10	.11	.11
**Chain B**									
1996	—	—		—	—	—	—	—	—
1997	—	370		380	730	550	204	280	—
1998	—	—		—	—	—	—	—	—
1999	250	400	590	360	760	530	204	280	407
2000	250	400	590	360	760	530	204	280	407
2001	230	360	500	370	780	550	—	—	—
2002	230	360	500	360	850	580	160	230	330
2003	230	360	500	360	800	580	160	230	330
2004	230	360	500	350	800	570	160	200	330
2005	230	360	500	350	800	570	140	200	290
2006	230	360	500	330	760	510	140	200	290
2007	230	360	500	330	760	510	140	200	290
2008	230	360	500	340	770	490	140	210	290
2009	340	480	580	340	770	490	210	290	390
2010	340	440	540	310	770	490	210	290	390
2011	340	440	540	300	760	470	190	290	380
2012	340	410	500	280	760	470	190	290	380
2013	340	410	500	288	710	510	190	290	380
**β**	8.9	3.9	−2.0	−5.8	−1.7	−5.5	−0.1	1.5	0.6
***P* value**	.001	.05	.36	<.001	.39	.002	.97	.52	.85
**Chain C**									
1996	260	380	460	320	490	310	90	108	180
1997	270	390	470	320	490	310	90	108	180
1998	270	470	570	320	490	310	90	108	180
1999	270	470	570	320	490	310	90	108	180
2000	270	470	570	320	490	310	90	108	180
2001	270	420	470	310	480	300	104	130	208
2002	250	390	440	310	480	300	112	140	224
2003	250	390	440	310	480	300	112	140	224
2004	250	390	440	310	480	360	112	140	224
2005	280	440	490	320	490	360	112	140	224
2006	370	420	540	320	490	370	140	224	294
2007	330	420	520	320	500	320	140	224	294
2008	340	430	550	260	500	320	140	210	280
2009	330	420	540	270	540	350	160	240	320
2010	330	410	540	270	540	340	160	240	320
2011	320	420	530	270	550	360	160	240	320
2012	320	420	530	290	620	390	160	—	—
2013	310	410	500	290	600	390	160	240	320
**β**	4.9	−0.5	1.8	−3.0	6.1	4.5	5.2	10.3	10.6
***P* value**	.001	.73	.40	<.001	<.001	<.001	<.001	<.001	<.001
**Chain comparison *P* value**	≤.002	.04^a^	NS	≤.008^b^	<.001^b^	<.001	≤.01	≤.02	<.001^b^

No one-time trend characterized the changes that occurred across chains. For example, the final energy content in 2013 of small French fries at chains A, B, and C was 20 kcal, 90 kcal, and 50 kcal higher, respectively, than in 1996 (all *P* ≤ .001) (Figure 1). For large French fries, the time trend changes were significant only for Chain A (*P* = .007), and for that chain the final energy content was 40 kcal lower in 2013 than in 1996. For cola we found a significant difference in time trends among the 3 restaurant chains (*P* ≤ .01).

#### Sodium

**Figure 2:**
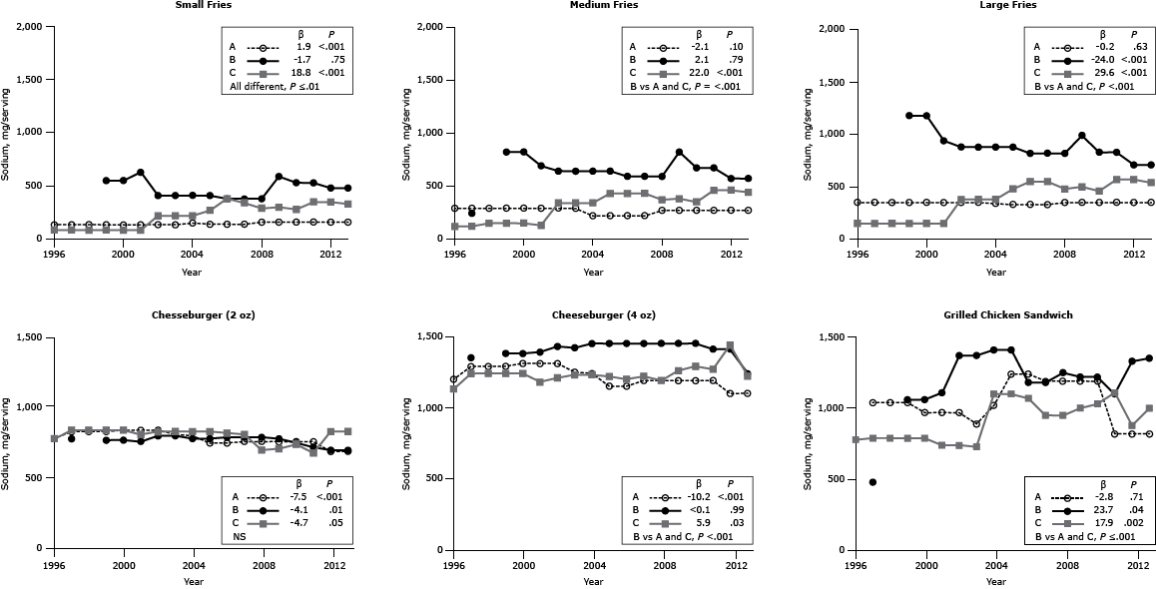
Sodium content (mg per portion) for popular menu items at 3 large, national fast-food chains. Sodium content for 3 sizes of French fries (small, medium, large), 2 sizes of cheeseburgers (2 oz, 4 oz), and 1 size of grilled chicken sandwich from Chains A, B, and C from 1996 through 2013. β estimates and *P *values derived from individual simple linear models, with energy as the dependent variable and time as the independent variable; chain comparison *P* values derived from ANOVA (analysis of variance) models comparing mean values between restaurants. Dashes indicate that data were not available; blank cells indicate that the item was not offered for the year(s). Abbreviation: NS, nonsignificant. ^a^ Difference is between Chain B versus Chains A and C. **Table:** 

Of the 18 items examined for sodium, the sodium content of 5 (27%) items decreased significantly (β range, −4.1 to −24.0 mg, *P* ≤ .05) (Figure 2). In contrast, the sodium content of 7 (39%) items significantly increased (β range, 1.9–29.6 mg, *P* ≤ .04). Average sodium content differed among chains for all individual menu items (*P* ≤ .01) except the 2-oz cheeseburger. We found marked heterogeneity among chains.

Chain/Year	French Fries			Cheeseburger		Grilled Chicken Sandwich
	Small	Medium	Large	2 oz	4 oz	
	Sodium, mg					
**Chain A**						
1996	135	290	350	770	1,200	
1997	135	290	350	820	1,290	1,040
1998	135	290	350	820	1,290	1,040
1999	135	290	350	820	1,290	1,040
2000	135	290	350	830	1,310	970
2001	135	290	350	830	1,310	970
2002	135	290	350	830	1,310	970
2003	135	290	350	800	1,250	890
2004	150	220	340	790	1,240	1,020
2005	140	220	330	740	1,150	1,240
2006	140	220	330	740	1,150	1,240
2007	140	220	330	750	1,190	1,190
2008	160	270	350	750	1,190	1,190
2009	160	270	350	750	1,190	1,190
2010	160	270	350	750	1,190	1,190
2011	160	270	350	750	1,190	820
2012	160	270	350	680	1,100	820
2013	160	270	350	680	1,100	820
**β**	1.9	−2.1	−0.2	−7.5	−10.2	−2.8
***P* value**	<.001	.10	.63	<.001	<.001	.71
**Chain B**						
1996	—	—		—	—	—
1997	—	240		770	1,350	480
1998	—	—		—	—	—
1999	550	820	1,180	760	1,380	1,060
2000	550	820	1,180	760	1,380	1,060
2001	630	690	940	750	1,390	1,110
2002	410	640	880	790	1,430	1,370
2003	410	640	880	790	1,420	1,370
2004	410	640	880	770	1,450	1,410
2005	410	640	880	770	1,450	1,410
2006	380	590	820	780	1,450	1,180
2007	380	590	820	780	1,450	1,180
2008	380	590	820	780	1,450	1,250
2009	590	820	990	770	1,450	1,220
2010	530	670	830	740	1,450	1,220
2011	530	670	830	710	1,410	1,100
2012	480	570	710	690	1,410	1,330
2013	480	570	710	690	1,240	1,350
**β**	−1.7	2.1	−24.0	−4.1	<0.1	23.7
***P* value**	.75	.79	<.001	.01	.99	.04
**Chain C**						
1996	85	120	150	770	1,130	780
1997	85	120	150	830	1,240	790
1998	85	150	180	830	1,240	790
1999	85	150	180	830	1,240	790
2000	85	150	180	830	1,240	790
2001	85	130	150	800	1,180	740
2002	220	340	380	820	1,210	740
2003	220	340	380	820	1,230	730
2004	220	340	380	820	1,230	1,100
2005	270	430	480	820	1,220	1,100
2006	380	430	550	810	1,200	1,070
2007	340	430	550	800	1,220	950
2008	290	370	480	690	1,190	950
2009	300	380	500	700	1,260	1,000
2010	280	350	460	730	1,290	1,030
2011	350	460	570	670	1,270	1,110
2012	350	460	570	820	1,440	880
2013	330	440	540	800	1,220	1,000
**β**	18.8	22.0	29.6	−4.7	5.9	17.9
***P* value**	<.001	<.001	<.001	.05	.03	.002
**Chain comparison *P* value**	≤.01	<.001^a^	<.001^a^	NS	<.001^a^	≤.001^a^

#### Saturated and *trans* fat

**Figure 3:**
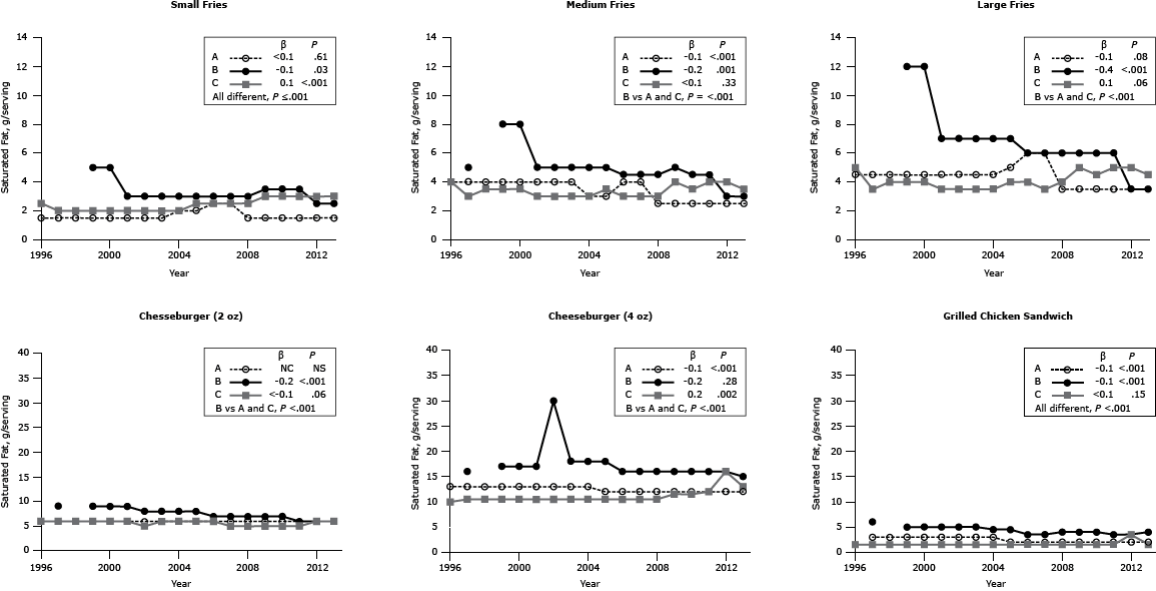
Saturated fat content (g per portion) for popular menu items at 3 large, national fast-food chains. Saturated fat content per serving for 3 sizes of French fries (small, medium, large), 2 sizes of cheeseburgers (2 oz, 4 oz), and 1 size of grilled chicken sandwich from Chains A, B, and C from 1996 through 2013. β estimates and *P* values derived from individual simple linear models; chain comparison *P* values derived from ANOVA (analysis of variance) models comparing mean values between restaurants. Dashes indicate that data were not available; blank cells indicate that the item was not offered for the year(s). Abbreviations: NS, nonsignificant; NC, no change. ^a^ Difference is between Chain B versus Chains A and C. **Table:** 

The saturated fat content of French fries, post-2001, was modest for all chains (1.5–6.0 g) (Figure 3). We found a noticeable decline in the saturated fat content of chain B’s French fries between 2000 and 2001. Nevertheless, the saturated fat content of the large-sized meal in 2013 contained 61% to 80% of the recommended 10% of energy upper limit (22 g/2,000 kcal) (1) and 104% and 135% of the recommended 6% of energy upper limit (13 g/2,000 kcal)(6).

Chain/Year	French Fries			Cheeseburger		Grilled Chicken Sandwich
	Small	Medium	Large	2 oz	4 oz	
	Saturated Fat, g					
**Chain A**						
1996	1.5	4.0	4.5	6.0	13.0	
1997	1.5	4.0	4.5	6.0	13.0	3.0
1998	1.5	4.0	4.5	6.0	13.0	3.0
1999	1.5	4.0	4.5	6.0	13.0	3.0
2000	1.5	4.0	4.5	6.0	13.0	3.0
2001	1.5	4.0	4.5	6.0	13.0	3.0
2002	1.5	4.0	4.5	6.0	13.0	3.0
2003	1.5	4.0	4.5	6.0	13.0	3.0
2004	2.0	3.0	4.5	6.0	13.0	3.0
2005	2.0	3.0	5.0	6.0	12.0	2.0
2006	2.5	4.0	6.0	6.0	12.0	2.0
2007	2.5	4.0	6.0	6.0	12.0	2.0
2008	1.5	2.5	3.5	6.0	12.0	2.0
2009	1.5	2.5	3.5	6.0	12.0	2.0
2010	1.5	2.5	3.5	6.0	12.0	2.0
2011	1.5	2.5	3.5	6.0	12.0	2.0
2012	1.5	2.5	3.5	6.0	12.0	2.0
2013	1.5	2.5	3.5	6.0	12.0	2.0
**β**	<0.1	−0.1	−0.1	NC	−0.1	−0.1
***P* value**	.61	<.001	.08	NS	<.001	<.001
**Chain B**						
1996	—	—		—	—	—
1997	—	5.0		9.0	16.0	6.0
1998	—	—		—	—	—
1999	5.0	8.0	12.0	9.0	17.0	5.0
2000	5.0	8.0	12.0	9.0	17.0	5.0
2001	3.0	5.0	7.0	9.0	17.0	5.0
2002	3.0	5.0	7.0	8.0	30.0	5.0
2003	3.0	5.0	7.0	8.0	18.0	5.0
2004	3.0	5.0	7.0	8.0	18.0	4.5
2005	3.0	5.0	7.0	8.0	18.0	4.5
2006	3.0	4.5	6.0	7.0	16.0	3.5
2007	3.0	4.5	6.0	7.0	16.0	3.5
2008	3.0	4.5	6.0	7.0	16.0	4.0
2009	3.5	5.0	6.0	7.0	16.0	4.0
2010	3.5	4.5	6.0	7.0	16.0	4.0
2011	3.5	4.5	6.0	6.0	16.0	3.5
2012	2.5	3.0	3.5	6.0	16.0	3.5
2013	2.5	3.0	3.5	6.0	15.0	4.0
**β**	−0.1	−0.2	−0.4	−0.2	−0.2	−0.1
***P* value**	.03	.001	<.001	<.001	.28	<.001
**Chain C**						
1996	2.5	4.0	5.0	6.0	10.0	1.5
1997	2.0	3.0	3.5	6.0	10.5	1.5
1998	2.0	3.5	4.0	6.0	10.5	1.5
1999	2.0	3.5	4.0	6.0	10.5	1.5
2000	2.0	3.5	4.0	6.0	10.5	1.5
2001	2.0	3.0	3.5	6.0	10.5	1.5
2002	2.0	3.0	3.5	5.0	10.5	1.5
2003	2.0	3.0	3.5	6.0	10.5	1.5
2004	2.0	3.0	3.5	6.0	10.5	1.5
2005	2.5	3.5	4.0	6.0	10.5	1.5
2006	2.5	3.0	4.0	6.0	10.5	1.5
2007	2.5	3.0	3.5	5.0	10.5	1.5
2008	2.5	3.0	4.0	5.0	10.5	1.5
2009	3.0	4.0	5.0	5.0	11.5	1.5
2010	3.0	3.5	4.5	5.0	11.5	1.5
2011	3.0	4.0	5.0	5.0	12.0	1.5
2012	3.0	4.0	5.0	6.0	16.0	3.5
2013	3.0	3.5	4.5	6.5	13.0	1.5
**β**	0.1	<0.1	0.1	<−0.1	0.2	<0.1
***P* value**	<.001	.33	.06	.06	.002	.15
**Chain comparison *P* value**	≤.001	<.001^a^	<.001^a^	<.001^a^	<.001^a^	<.001

**Figure 4:**
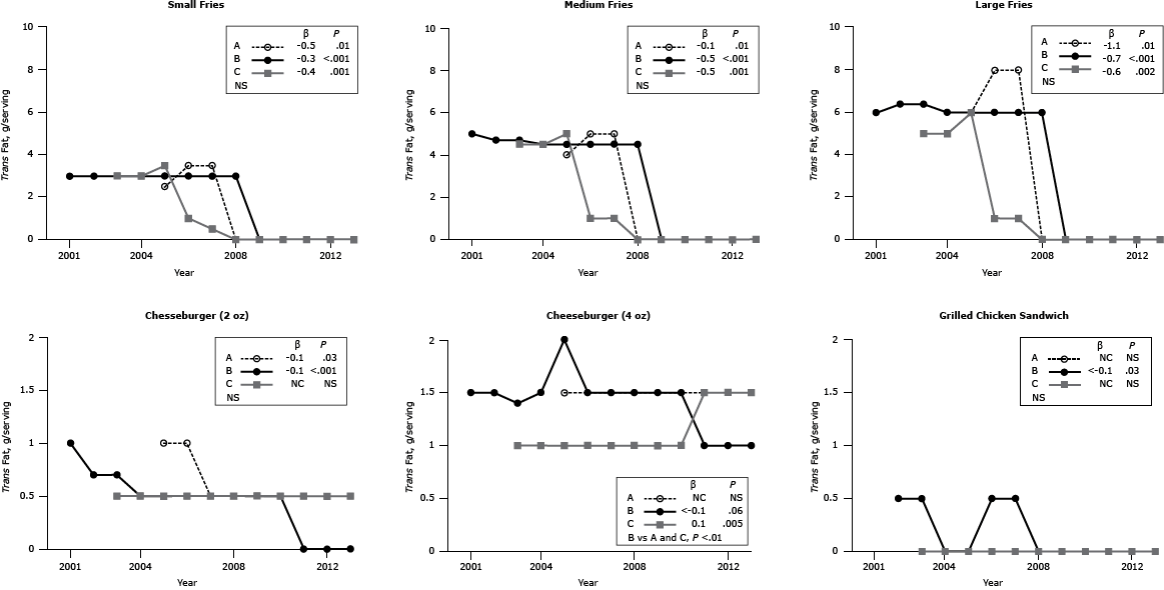
*Trans* fat content (g per portion) for popular menu items at 3 large, national fast-food chains. *Trans* fat content per serving for 3 sizes of French fries (small, medium, large), 2 sizes of cheeseburgers (2 oz, 4 oz), and 1 size of grilled chicken sandwich from Chains A, B, and C from 2001 through 2013. β estimates and *P* values derived from individual simple linear models; chain comparison *P* values derived from ANOVA (analysis of variance) models, comparing mean values between restaurants. Dashes indicate that data were not available; blank cells indicate that the item was not offered for the year(s). ^a^ Difference is between Chain C versus Chains A and B. Abbreviations: NS, nonsignificant; NC, no change. **Table:** 

Data for *trans* fat became available in 2001. The *trans* fat content of French fries declined to undetectable levels between 2006 and 2009 (Figure 4).

Chain/Year	French Fries			Cheeseburger		Grilled Chicken Sandwich
	Small	Medium	Large	2 oz	4 oz	
	*Trans* Fat, g					
**Chain A**						
2001	—	—	—	—	—	—
2002	—	—	—	—	—	—
2003	—	—	—	—	—	—
2004	—	—	—	—	—	—
2005	2.5	4.0	6.0	1.0	1.5	0
2006	3.5	5.0	8.0	1.0	1.5	0
2007	3.5	5.0	8.0	0.5	1.5	0
2008	0	0	0	0.5	1.5	0
2009	0	0	0	0.5	1.5	0
2010	0	0	0	0.5	1.5	0
2011	0	0	0	0.5	1.5	0
2012	0	0	0	0.5	1.5	0
2013	0	0	0	0.5	1.5	0
**β**	−0.5	−0.1	−1.1	−0.1	NC	NC
***P* value**	.01	.01	.01	.03	NS	NS
**Chain B**						
2001	3.0	5.0	6.0	1.0	1.5	—
2002	3.0	4.7	6.4	0.7	1.5	0.5
2003	3.0	4.7	6.4	0.7	1.4	0.5
2004	3.0	4.5	6.0	0.5	1.5	0
2005	3.0	4.5	6.0	0.5	2.0	0
2006	3.0	4.5	6.0	0.5	1.5	0.5
2007	3.0	4.5	6.0	0.5	1.5	0.5
2008	3.0	4.5	6.0	0.5	1.5	0
2009	0	0	0	0.5	1.5	0
2010	0	0	0	0.5	1.5	0
2011	0	0	0	0	1.0	0
2012	0	0	0	0	1.0	0
2013	0	0	0	0	1.0	0
**β**	−0.3	−0.5	−0.7	−0.1	<−0.1	<−0.1
***P* value**	<.001	<.001	<.001	<.001	.06	.03
**Chain C**						
2001	—	—	—	—	—	—
2002	—	—	—	—	—	—
2003	3.0	4.5	5.0	0.5	1.0	0
2004	3.0	4.5	5.0	0.5	1.0	0
2005	3.5	5.0	6.0	0.5	1.0	0
2006	1.0	1.0	1.0	0.5	1.0	0
2007	0.5	1.0	1.0	0.5	1.0	0
2008	0	0	0	0.5	1.0	0
2009	0	0	0	0.5	1.0	0
2010	0	0	0	0.5	1.0	0
2011	0	0	0	0.5	1.5	0
2012	0	0	0	0.5	1.5	0
2013	0	0	0	0.5	1.5	0
**β**	−0.4	−0.5	−0.6	NC	0.1	NC
***P* value**	.001	.001	.002	NS	.005	NS
**Chain comparison *P* value**	NS	NS	NS	NS	<.01^a^	NS

### Meals

**Figure 5:**
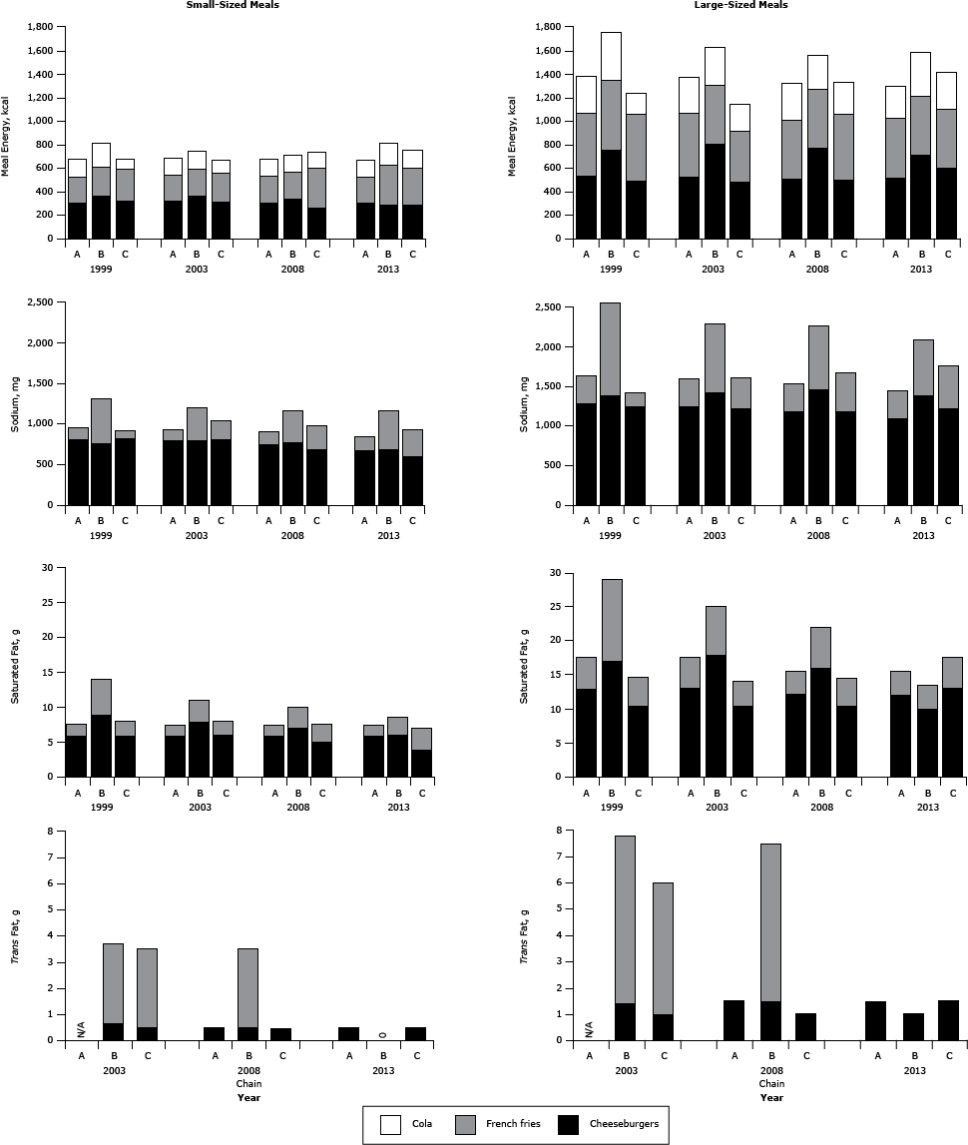
Comparison of energy, sodium, saturated fat, and *trans* fat content for popular menu items at 3 large, national fast-food chains. Energy (kcal), sodium (mg), saturated fat (g), and *trans* fat (g) content for 1999, 2003, 2008, and 2013 in popular small-sized (ie, 2-oz cheeseburger, small French fries, and small cola) and large-sized (ie, 4-oz cheeseburger, large French fries, and large cola) meals from chains A, B, and C. *Trans* fat data were not available for 1999. Dashes indicate that data were not available. Abbreviation: NA, not applicable. **Table:** 

Over time the total energy content of the bundled meal varied inconsistently among the 3 chains (Figure 5). In 2013, the energy content of a large-sized bundled meal (cheeseburger, French fries, and regular cola) represented 65% to 80% of a 2,000-calorie-per-day diet, and sodium content represented 63% to 91% of the 2,300-mg-per-day recommendation and 97% to 139% of the 1,500-mg-per-day recommendation. We found a gradual downward trend in the sodium content of Chain A and Chain B and a gradual upward trend for Chain C. The saturated fat content of the meals exhibited little change for Chain A or Chain C, whereas it steadily declined for Chain B. The total *trans* fat content of the combination meals declined dramatically.

Year/Item	Chain A	Chain B	Chain C
**Energy, kcal (% Total meal kcal)**			
**1999**			
2-oz Cheeseburger	320 (47)	360 (44)	320 (47)
Small French fries	210 (31)	250 (31)	270 (40)
Small cola	150 (22)	204 (25)	90 (13)
Total small meal	680 (100)	814 (100)	680 (100)
4-oz Cheeseburger	530 (38)	760 (43)	490 (40)
Large French fries	540 (39)	590 (34)	570 (46)
Large cola	310 (22)	407 (23)	180 (15)
Total large meal	1,380 (100)	1,757 (100)	1,240 (100)
**2003**			
2-oz Cheeseburger	330 (48)	360 (48)	310 (46)
Small French fries	210 (30)	230 (31)	250 (37)
Small cola	150 (22)	160 (21)	112 (17)
Total small meal	690 (100)	750 (100)	672 (100)
4-oz Cheeseburger	530 (38)	800 (49)	480 (42)
Large French fries	540 (39)	500 (31)	440 (38)
Large cola	310 (22)	330 (20)	224 (20)
Total large meal	1,380 (100)	1,630 (100)	1,144 (100)
**2008**			
2-oz Cheeseburger	300 (44)	340 (48)	260 (35)
Small French fries	230 (34)	230 (32)	340 (46)
Small cola	150 (22)	140 (20)	140 (19)
Total small meal	680 (100)	710 (100)	740 (100)
4-oz Cheeseburger	510 (39)	770 (49)	500 (38)
Large French fries	500 (38)	500 (32)	550 (41)
Large cola	310 (23)	290 (19)	280 (21)
Total large meal	1,320 (100)	1,560 (100)	1,330 (100)
**2013**			
2-oz Cheeseburger	300 (45)	288 (35)	290 (38)
Small French fries	230 (34)	340 (42)	310 (41)
Small cola	140 (21)	190 (23)	160 (21)
Total small meal	670 (100)	818 (100)	760 (100)
4-oz Cheeseburger	520 (40)	710 (45)	600 (42)
Large French fries	500 (38)	500 (31)	500 (35)
Large cola	280 (22)	380 (24)	320 (23)
Total large meal	1,300 (100)	1,590 (100)	1,420 (100)
**Sodium, mg (% of total meal)**			
**1999**			
2-oz Cheeseburger	820 (86)	760 (58)	830 (91)
Small French fries	135 (14)	550 (42)	85 (9)
Total small meal	955 (100)	1,310 (100)	915 (100)
4-oz Cheeseburger	1,290 (79)	1,380 (54)	1,240 (87)
Large French fries	350 (21)	1,180 (46)	180 (13)
Total large meal	1,640 (100)	2,560 (100)	1,420 (100)
**2003**			
2-oz Cheeseburger	800 (86)	790 (66)	820 (79)
Small French fries	135 (14)	410 (34)	220 (21)
Total small meal	935 (100)	1,200 (100)	1,040 (100)
4-oz Cheeseburger	1,250 (78)	1,420 (62)	1,230 (76)
Large French fries	350 (22)	880 (38)	380 (24)
Total large meal	1,600 (100)	2,300 (100)	1,610 (100)
**2008**			
2-oz Cheeseburger	750 (82)	780 (67)	690 (70)
Small French fries	160 (18)	380 (33)	290 (30)
Total small meal	910 (100)	1,160 (100)	980 (100)
4-oz Cheeseburger	1,190 (77)	1450 (64)	1,190 (71)
Large French fries	350 (23)	820 (36)	480 (29)
Total large meal	1,540 (100)	2,270 (100)	1,670 (100)
**2013**			
2-oz Cheeseburger	680 (81)	690 (59)	600 (65)
Small French fries	160 (19)	480 (41)	330 (35)
Total small meal	840 (100)	1,170 (100)	930 (100)
4-oz Cheeseburger	1,100 (76)	1,380 (66)	1,220 (69)
Large French fries	350 (24)	710 (34)	540 (31)
Total large meal	1,450 (100)	2,090 (100)	1,760 (100)
**Saturated fat, g (% total meal)**			
**1999**			
2-oz Cheeseburger	6 (80)	9 (64)	6 (75)
Small French fries	1.5 (20)	5 (36)	2 (25)
Total small meal	7.5 (100)	14 (100)	8 (100)
4-oz Cheeseburger	13 (74)	17 (59)	10.5 (72)
Large French fries	4.5 (26)	12 (41)	4 (28)
Total large meal	17.5 (100)	29 (100)	14.5 (100)
**2003**			
2-oz Cheeseburger	6 (80)	8 (73)	6 (75)
Small French fries	1.5 (20)	3 (27)	2 (25)
Total small meal	7.5 (100)	11 (100)	8 (100)
4-oz Cheeseburger	13 (74)	18 (72)	10.5 (75)
Large French fries	4.5 (26)	7 (28)	3.5 (25)
Total large meal	17.5 (100)	25 (100)	14 (100)
**2008**			
2-oz Cheeseburger	6 (80)	7 (70)	5 (67)
Small French fries	1.5 (20)	3 (30)	2.5 (33)
Total small meal	7.5 (100)	10 (100)	7.5 (100)
4-oz Cheeseburger	12 (77)	16 (73)	10.5 (72)
Large French fries	3.5 (23)	6 (27)	4 (28)
Total large meal	15.5 (100)	22 (100)	14.5 (100)
**2013**			
2-oz Cheeseburger	6 (80)	6 (71)	4 (57)
Small French fries	1.5 (20)	2.5 (29)	3 (43)
Total small meal	7.5 (100)	8.5 (100)	7 (100)
4-oz Cheeseburger	12 (77)	10 (74)	13 (74)
Large French fries	3.5 (23)	3.5 (26)	4.5 (26)
Total large meal	15.5 (100)	13.5 (100)	17.5 (100)
***Trans* fat, g (% total meal)**			
**2003**			
2-oz Cheeseburger	—	0.7 (19)	0.5 (14)
Small French fries	—	3 (81)	3 (86)
Total small meal	—	3.7 (100)	3.5 (100)
4-oz Cheeseburger	—	1.4 (18)	1 (17)
Large French fries	—	6.4 (82)	5 (83)
Total large meal	—	7.8 (100)	6 (100)
**2008**			
2-oz Cheeseburger	0.5 (100)	0.5 (14)	0.5 (100)
Small French fries	0	3 (86)	0
Total small meal	0.5 (100)	3.5 (100)	0.5 (100)
4-oz Cheeseburger	1.5 (100)	1.5 (20)	1 (100)
Large French fries	0	6 (80)	0
Total large meal	1.5 (100)	7.5 (100)	1 (100)
**2013**			
2-oz Cheeseburger	0.5 (100)	0	0.5 (100)
Small French fries	0	0	0
Total small meal	0.5 (100)	0	0.5 (100)
4-oz Cheeseburger	1.5 (100)	1 (100)	1.5 (100)
Large French fries	0	0	0
Total large meal	1.5 (100)	1 (100)	1.5 (100)

## Discussion

Despite concern that portion sizes have increased over time, contributing to the obesity epidemic and high rates of cardiometabolic disorders, among the 3 fast-food chain restaurants surveyed and menu items selected, no clear temporal trends were observed. Nevertheless, the energy, sodium, and saturated fat contents were high relative to recommendations (1).

Although there appeared to be an upward trend in portion size of the fast-food restaurant items through 2002 (13), our data and recently published data (17) indicate that this trend appears to have abated. Changes did vary substantially among the 3 chains, and portion sizes remain large. For example, in 2013, large-sized fries, regardless of chain, represented 25% of the daily energy needs of an adult, assuming an energy requirement of 2,000 kcal per day. A large-sized bundled meal composed of a large cheeseburger, large French fries, and regular cola beverage represented 65% to 80% of a 2,000 calorie diet.

Although the sodium content of some menu items decreased, sodium levels mostly remain high and in some cases they increased. The Dietary Guidelines for Americans recommends a sodium limit of 2,300 mg per day for the general population and 1,500 mg per day for some subpopulations (1). For all 3 chains, the sodium content of a 4-oz cheeseburger approached or exceeded half the 2,300-mg-per-day target and 75% of the 1,500-mg-per-day target (1). For the large-sized meal, the sodium content was 63% to 91% of a 2,300-mg-per-day recommendation and 97% to 139% of a 1,500-mg-per-day recommendation.

The saturated fat content of the items surveyed was consistent, with the exception of a decrease in 1 chain’s French fries, presumably due to a change in frying fat (from beef tallow to partially hydrogenated fat). Nevertheless, the saturated fat content of the large-sized meal in 2013 was 61% to 80% of the recommended 10% of energy upper limit (22 g/2,000 kcal) (1) and 104% and 135% of the recommended 6% of energy upper limit (13 g/2,000 kcal)(6).

Of the menu items assessed, French fries historically contributed most of the *trans* fat. More recently, the *trans* fat content of French fries decreased because of a shift away from the use of partially hydrogenated fat. This change was prompted by local legislative mandates and public pressure (16). Our findings are consistent with those recently generated for foods purchased from 11 fast-food chains in New York City (18). The changes are an example of how reformulation had a positive effect on overall intake (19). The American Heart Association recommends *trans* fat intake be less than 1% of energy (20). The *trans* fat content of the large bundled meal that includes a cheeseburger represents 50% to 75% of the current recommendation, although the *trans* fat content of cheeseburgers comes from that naturally present in ruminant fat.

A noteworthy finding was that the energy, sodium, and saturated fat content of similarly labeled menu items differed considerably among the 3 fast-food chains. This has implications for counseling individuals on approaches to reduce intakes. Although the most straightforward approach is to provide dietary counseling on the basis of portion size (eg, always order the smallest size), findings of our study indicate that without information tailored to each food venue, the counseling is not likely to achieve the intended goal. To illustrate, an order of small French fries at Chain B provides 110 kcal and 320 mg sodium more than the same item at Chain A. It is unlikely that consumers are aware of the differences among chain restaurants. However, the implications of these data are striking. An extra 100 kcal per day without compensation translates to a 6 to 7 kg weight gain per year (21–23).

The data in this study were restricted to 3 fast-food chain restaurants; therefore, trends observed may not be generalizable to other venues. Of note, the 3 fast-food chain restaurants chosen account for approximately 34% of sales dollars of the top fast-food restaurants in the Untied States (14). Additionally, fast-food chain restaurant foods and beverages accounted for approximately 40% of total away-from-home energy intake in 2008 (24). Consumption of foods and beverages from fast-food restaurants is positively associated with body fatness and coronary heart disease mortality (25–28). Not captured in this study was the potential effect of recent “healthier” items offered by fast-food restaurants on the choice of the more frequently ordered items that were the focus of this study.

Most of the data used for this study were derived from the Wayback Machine website. When possible, these data were independently validated using current data from company websites. In all cases, the Wayback Machine website data were accurate. This website is unbiased because it is an independent website archival system. We cannot rule out the possibility that small variations in nutrient values from year to year may have resulted from analytical variability or shifts in analytical methods. The study was limited to 3 chain restaurants, 3 food items, 1 beverage, and 1 bundled meal. These items and this meal combination were chosen because they were most commonly ordered in the 3 chain restaurants (15). An alternate approach would have been to choose different restaurants on the basis of single items, for example, chicken sandwiches. Although trends for other food items or other types of fast-food items could be different, there are no data to suggest this to be the case. An unanswered question is whether, were a fast-food chain to change its portion sizes or reduce sodium content, consumers would compensate by modifying their order, switching to another chain, or altering another dietary component.

Our findings suggest that efforts to promote reductions in energy, sodium, saturated fat, and *trans* fat intakes need to be shifted from emphasizing portion size labels (eg, small, medium, large). When developing strategies that help consumers better control their energy intakes and intakes of other nutrients, additional factors — such as total caloric intake, frequency of eating occasions, number of items eaten at any occasion, specific menu choices, and limiting energy-containing beverages — should be addressed. People should be encouraged to take advantage of the point-of-purchase menu labeling provided at fast-food establishments and should consult websites that contain nutrition information.
